# Comparison of ruminal ecology and blood profiles in Bali, Madura, and Ongole crossbred cattle of Indonesia

**DOI:** 10.14202/vetworld.2025.379-387

**Published:** 2025-01-15

**Authors:** Yenny Nur Anggraeny, Peni Wahyu Prihandini, Mozart Nuzul Aprilliza, Yeni Widiawati, Dicky Pamungkas, Mariyono Mariyono, Noor Hudhia Krishna, Risa Antari, Setiasih Setiasih, Bess Tiesnamurti, Muhammad Nasir Rofiq, Windu Negara, Eni Siti Rohaeni, Firsoni Firsoni, Wahidin Teguh Sasongko

**Affiliations:** 1 Research Center for Animal Husbandry, Research Organization for Agriculture and Food, National Research and Innovation Agency of The Republic of Indonesia, Bogor 16911, Indonesia; 2 Research Center for Sustainable Production System and Life Cycle Assessment, Research Organization for Energy and Manufacturing, National Research and Innovation Agency, Banten 15314, Indonesia

**Keywords:** Bali cattle, blood profile, hematology, Madura cattle, Ongole crossbred cattle, rumen fluid, volatile fatty acids

## Abstract

**Background and Aim:**

Indonesian cattle breeds, primarily Bali, Madura, and Ongole crossbred (OC), are vital to local farming systems, yet little is known about their ruminal ecology and blood profiles. This study aimed to compare the rumen fluid characteristics and hematological parameters among these three indigenous cattle breeds.

**Materials and Methods:**

Thirty heifers (10 per breed) were sourced from the Indonesian Beef Cattle Research Station. The animals, weighing 175–197 kg, were randomly allocated to individual pens. A diet of commercial concentrate and elephant grass (70:30 ratio) was provided at 3.5% of their body weight (dry matter basis). Blood samples were analyzed for glucose, blood urea nitrogen (BUN), and hematological indices (White blood cell, red blood cells [RBC], hemoglobin, hematocrit, mean corpuscular hemoglobin [MCH], and mean corpuscular volume [MCV]). Rumen fluid was assessed for pH, NH_3_, volatile fatty acids (VFAs), and microbial diversity. Statistical analyses were performed using the Statistical Package for the Social Sciences with significance set at p < 0.05.

**Results:**

No significant differences were observed in blood glucose and BUN levels across breeds. Bali cattle exhibited the highest concentrations of total VFAs (139.66 mMol) and propionic acid (33.31 mMol), with a lower acetic-to-propionic acid ratio, reflecting efficient glucogenic traits. *Quinella*, a propionate-producing bacterium, dominated Bali cattle rumen microbiota. Conversely, OC cattle demonstrated the highest RBC count (9.27 x 10³/µL), while Bali cattle showed superior RBC size (MCV: 48.84 fl) and hemoglobin content (MCH: 16.60 pg).

**Conclusion:**

Bali cattle exhibited superior rumen fermentation efficiency and favorable hematological profiles, potentially supporting enhanced productive performance and reduced enteric methane emissions. These findings provide a foundation for breed-specific dietary management strategies to optimize local cattle productivity in tropical environments.

## INTRODUCTION

Tropical beef cattle originate from regions of the world with tropical climates [1]. *Bos indicus* (Zebu cattle) is a tropical beef cattle breed with a hump [2, 3]. *B. indicus* cattle can live in high-temperature environments, utilize low-quality feed ingredients, and are resistant to internal and external parasites [4]. In Indonesia, most of the native cattle breeds are descended from the *B. indicus* and *Bos javanicus* breeds [5]. The *B. indicus* cattle breed examples are the Ongole crossbred (OC), Madura, Java, Sumatran cattle, and other indigenous cattle, while the *B. javanicus* breed is Bali cattle [6, 7]. Smallholder farmers mostly keep three Indonesian local cattle breeds: OC, Bali, and Madura cattle [7]. Indonesian cattle breeds are kept and used by farmers for draught animals, feeder beef, and organic fertilizers [8, 9]. Local Indonesian cattle have diverse genetics from *B. indicus* and *B. javanicus*, which can adapt to low-quality feed and can be reared with traditional extensive rearing systems, as well as resistant to several diseases, parasites, and heat stress [3, 4, 10, 11].

In Indonesia, OC, Bali, and Madura cattle are commonly fed with native grass that provides low-quality nutrition. In ruminants, rumen microbes play several roles, including feeding degraders [12, 13] and sources of protein for the host ruminants [14, 15]. Previous studies by Daghio *et al*. [16] and Marcos *et al*. [17] have reported that cattle breeds can affect the microbiota community in the rumen even when they eat the same feed. Rumen microbes are influenced by breed, rumen fermentation, and feed type. In traditional cattle rearing, forage is the main feed for cattle, and it greatly affects rumen fermentation. In the rumen, feed undergoes fermentation and decomposition by enzymes produced by the anaerobic microbes of rumen fluid. The fermentation process in rumen produces various end-products, such as volatile fatty acids (VFA) and ammonia (NH_3_) [18]. Diet can affect absorptive metabolism in rumen epithelial tissue. Diets containing high concentrations of concentrate have previously been shown to increase total VFA content in the rumen [19]. In addition, high concentration feeding increased propionate and butyrate levels [20, 21]. The main components of VFA that are fermented in rumen are acetic acid, propionic acid, and butyric acid [22].

Feeds affect rumen fermentation and blood parameters, which have a positive impact on cattle performance. Blood plays a role in transporting nutrients such as vitamins, minerals, glucose, fats, and proteins throughout the body. Blood profile examination is very important because blood has a vital function in all living things and helps monitor the incidence of diseases. The blood profile is useful for assessing health conditions and as a reference or control in a study due to metabolic disorders, diseases, damage to organ structures or functions, the influence of agents or drugs, and stress [23, 24]. Changes in blood profiles can also occur when there is feed restriction, such as when local cattle are raised traditionally by farmers [8, 25, 26]. Some biochemical tests of blood and other body fluids in livestock can be used to explain the mechanism of the occurrence of irregularities, provide an overview of health conditions and metabolic status, and help establish the diagnosis so that appropriate treatment can be given [27].

Therefore, it is significant to explore more research on rumen metabolism and blood profile of OC, Bali, and Madura cattle, which have not been extensively studied. This study aimed to investigate the ruminal ecology and blood profiles of three crossbred Indonesian cattle namely OC, Bali, and Madura cattle by analyzing their rumen fermentation characteristics, microbial diversity, and blood parameters to better understand their metabolic efficiency and potential implications for improving local cattle production systems.

## MATERIALS AND METHODS

### Ethical approval

The study was conducted under the guidelines of the Indonesian Code of Practice for the Care and Use of Animals for Scientific Purposes and was approved by the Indonesian Ministry of Agriculture Animal Ethics Committee (Balitbangtan/Lolitsapi/Rm/04/2021).

### Study period and location

**Figure 1:**
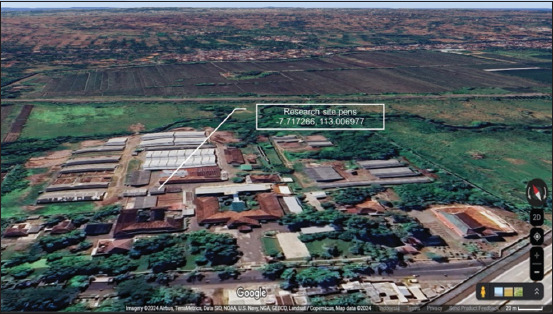
The location of the research was at the Indonesian Beef Cattle Research Station in Grati District, Pasuruan Regency, East Java Province, Indonesia.

This research was carried out during May and June 2021 in an experimental pen of Indonesian Beef Cattle Research Station, Grati, Pasuruan, East Java, Indonesia (Figure 1), with an elevation 25–100 m above sea level. The average temperature was 22.1°C, a range of 12.9–28.6°C, whereas the average humidity was 88%, with a range of 52%–82%. The average wind speed was 4.7 m/s, and the average atmospheric pressure was 83 mb in the 73.7–90.6 mb range. The total rainfall was 15.9 mm, and the average sun exposure was 58.9%.

### Experimental design

The experimental design was a randomized block design in which the initial weight was used as blocks with ten replicates. Three different indigenous cattle breeds were examined and compared in this study. The average body weight ± standard deviation of the OC, Bali, and Madura heifers were 196.85 ± 34.30 kg, 175.1 ± 58.59 kg, and 188 ± 34.50 kg, respectively. They were randomly housed in individual pens and had free access to fresh drinking water at all times.

The feed offered to the cattle consisted of commercial concentrate and elephant grass (*Pennisetum purpureum*) (Table 1). The concentrate: forage ratio was 70:30. The feed offered was 3.5% body weight on a dry matter basis. The concentrate was given at 7.30 am, while forage was given at 11.00 am.

**Table 1:** Nutrient content of feed given to livestock during the study.

No.	Feed type	DM (%)	CP	EE	CF	Ash	TDN	NDF

			(% DM)					
1.	Concentrate	88.58	18.13	6.26	15.53	9.96	70.12	34.01
2.	*Penisetum purpureum*	37.31	6.60	3.02	32.18	11.04	50.75	65.54

DM=Dry matter, CP=Crude protein, EE=Extract ether, CF=Crude fiber, TDN=Total digestible nutrient, NDF=Neutral detergent fiber

### Blood sampling and analysis

Blood (10 mL) was collected from the jugular vein at 4 h after feeding. The samples were analyzed for blood metabolites (glucose and blood urea nitrogen [BUN]) using the clinical chemistry analyzer (Boeki TRX 7010, Japan) and hematology (white blood cells, red blood cells [RBC], hemoglobin, hematocrit, mean corpuscular hemoglobin [MCH], and mean corpuscular volume [MPV] using a hematology auto-analyzer (Mindray BC-5000, China).

### Rumen fluid analysis

Rumen fluid samples were taken orally by trained casual workers, and they were about 50–75 mL. We used the samples to observe pH, NH_3_, VFA (acetic, propionic, butyric, isobutyric, valeric, and isovaleric acids), and protozoa population. The pH was measured using a digital pH meter (Hanna Hi 98107, Hanna Instruments, USA). The NH_3_ levels were determined using the indophenol method in a spectrophotometer (Thermo Scientific, USA). The individual VFA levels were examined using gas chromatography (Thermo Scientific). Rumen bacteria diversity was assessed as described by Caporaso *et al*. [28].

### Statistical analysis

The data collected in this study were subjected to a one-way analysis of variance using the Statistical Package for the Social Sciences (SPSS) version 16.0 (IBM SPSS Statistics, NY, USA) to determine the effects of breed on ruminal characteristics, blood profiles, and other measured parameters [29]. Differences among group means were assessed using Duncan’s Multiple Range Test (DMRT) at a significance level of p < 0.05. The results are presented as means ± standard deviations (SD).

## RESULTS

The blood glucose and BUN levels in the OC, Bali, and Madura cattle are shown in Table 2. Neither glucose nor BUN levels differed between the three breeds of cattle.

**Table 2:** Profile of blood serum metabolites of OC, Bali, and Madura heifers.

Variable	Mean			p-value

	OC	Bali	Madura	
Glucose (mg/dL)	56.70 ± 8.99	42.70 ± 16.99	58.20 ± 21.83	0.094
Blood Urea nitrogen (mg/dL)	46.50 ± 6.92	45.80 ± 3.12	41.70 ± 5.46	0.119

OC=Ongole crossbred

The characteristics of the rumen are presented in Table 3. There were differences in the concentrations of acetic acid (p = 0.030), propionic acid (p = 0.009), and total VFA (p = 0.028) among the three breeds. The same pattern occurred in the concentrations of acetic acid, propionic acid, and total VFA, where the highest concentration was in Bali cattle, followed by Madura and OC cattle. Interestingly, the populations of rumen protozoa did not differ significantly; this result is consistent with the normal pH in all groups. The highest total VFA concentration was observed in Bali cattle and the lowest was observed in OC cattle. The propionic acid concentration in Bali cattle was the highest than Madura and OC cattle, causing the ratio between C_2_ and C_3_ in Bali cattle to be lower than in Madura and OC cattle.

**Table 3:** Profiles of the rumen characteristics of OC, Bali, and Madura heifers.

Variable	Mean			p-value

	OC	Bali	Madura	
pH	6.92 ± 0.13	6.56 ± 0.15	6.78 ± 0.14	0.209
Protozoa (log 10/mm^3^)	6.50 ± 0.21	6.61 ± 0.20	6.44 ± 0.42	0.472
NH_3_ (mg/100 mL)	5.69 ± 1.29	5.69 ± 0.41	7.65 ± 2.60	0.075
Acetic acid (mMol)	62.41 ± 17.86^a^	86.84 ± 19.20^b^	69.15 ± 22.42^ab^	0.030
Propionic acid (mMol)	21.98 ± 6.93^a^	33.31 ± 7.36^b^	24.26 ± 9.52^a^	0.009
Butyric acid (mMol)	13.95 ± 4.09	17.02 ± 5.19	14.14 ± 4.83	0.281
Isobutyric acid (mMol)	0.55 ± 0.20	0.60 ± 0.27	0.62 ± 0.20	0.789
Valerat (mMol)	0.98 ± 0.25	1.17 ± 0.39	0.89 ± 0.24	0.132
Isovalerat (mMol)	0.61 ± 0.17	0.73 ± 0.34	0.61 ± 0.15	0.450
VFA total (mMol)	100.48 ± 29.07	139.66 ± 31.04	109.66 ± 36.5	0.028
Acetic acid: Propionic acid	2.87 ± 0.22	2.63 ± 0.32	2.97 ± 0.58	0.174

^a,b^Different superscripts in the same row indicate differences. VFA=Volatile fatty acids. OC=Ongole crossbred

The blood cell profiles of the OC, Bali, and Madura cattle are presented in Table 4. The breed of the cattle had a significant influence (p < 0.05) on the blood cell profile. The highest RBC counts were observed in OC cattle, while in Bali and Madura cattle, the RBC counts were similar. The OC cattle had higher hematocrit values than the Madura and Bali cattle. The Bali cattle had the highest MCH value. Meanwhile, Madura cattle had higher MCV values than Bali and OC cattle.

**Table 4:** Profile of blood cells of OC, Bali, and Madura breed.

Variable	Mean			p-value

	OC	Bali	Madura	
WBC (10^3^/µL)	10.39 ± 2.62	8.53 ± 2.11	10.09 ± 2.59	0.211
RBC (10^3^/µL)	9.27 ± 1.01^b^	8.31 ± 0.57^a^	8.23 ± 0.80^a^	0.014
Hb (g/dL)	12.00 ± 0.77	13.85 ± 1.39	11.51 ± 3.92	0.097
Hematocrit (%)	32.08 ± 6.92^a^	40.73 ± 4.38^b^	36.59 ± 3.17^ab^	0.003
MCH (pg)	13.03 ± 0.89^a^	16.60 ± 2.00c	15.22 ± 1.14^b^	0.000
MCV (fl)	37.28 ± 3.07^a^	48.84 ± 6.22^b^	44.61 ± 3.30c	0.000

^a,b,c^Different superscripts in the same row indicate differences. WBC=White blood cell, RBC: Red blood cell, Hb=Hemoglobin, MCH=Mean corpuscular hemoglobin, MCV=Mean corpuscular volume, OC=Ongole crossbred

## DISCUSSION

### Blood metabolites

Glucose is an important nutrient for several body tissues. Glucose in ruminants is obtained through the metabolism of propionic acid and amino acids [30, 31]. Statistically, blood glucose levels in the three breeds of local Indonesian cattle were not different. Shaffer *et al*. [32] also found that the breed of cattle had no effect on blood glucose levels in dairy cows. However, blood glucose levels in goats and chickens are affected by breed [33, 34]. The blood glucose level of Bali cattle in this study was slightly lower, around 42.70 mg/dL, compared with the results reported by Suharti *et al*. [35]. Our result of serum glucose in Madura cattle was lower than the result shown by Tombuku *et al*. [36], accounting for 69.96–73.28 mg/dL; however, the glucose level for OC cattle was lower compared with a previous study by Jusman *et al*. [37] (64.01 ± 7.90 mg/dL).

The liver produces BUN during the urea cycle as a waste product of protein digestion. The concentration of BUN in Bali cattle in the present study was higher than in another study [35], which was 16.10–18.97 mg/dL. BUN in Madura cattle was still within the recommendation range (35–50 mg/dL) reported By Umar *et al*. [38] but was higher than the results (25.03 mg/dL) reported by Fretas *et al*. [39]. The BUN of OC cattle in this study was higher than that reported by Widayati *et al*. (16.97–27.60 mg/dL) [40]. BUN concentration correlates with ammonia concentration in rumen and can indicate the adequacy of livestock’s energy intake. The efficiency of NH_3_ use for protein synthesis in rumen depends on the availability of energy. If energy restriction occurs, the protein will be excessive and cannot be used by rumen microbes, causing an increase in the concentration of urea in plasma [41].

### Rumen characteristics

The condition of the rumen determines the optimal process of feed digestion. Rumen microbes play a key role in the digestive process of ruminants. The rumen fluid profiles of the OC, Bali, and Madura cattle are presented in Table 3.

The rumen fluid pH values of the three breed cattle were not significantly different. However, our results are slightly lower than those reported by Bharanidharan *et al*. [42] and Carvalho and Felix [43], in which the pH was not affected by breed but was more likely to respond differently to feeding regimes [42, 43]. Purbowati *et al*. [44] maintained the pH of the rumen fluid around 6.8 through the regulation of the absorption of fatty acids and ammonia. The pH of the rumen fluid varies according to the type of feed. Rumen pH is a factor that affects the microbial population in rumen. The pH of rumen fluid of Madura and OC cattle in this study was lower than the results obtained by Bharanidharan *et al*. (7.9) [42].

The breed of the cattle did not affect the rumen fluid NH_3_ concentration, but the concentration in this study was sufficient for the needs of microbial protein synthesis at 3–8 mg/100 mL [45]. A previous study by Bharanidharan *et al*. [42] also reported that the concentration of NH_3_ was not affected by the breed of the cows. The protein degradation of feedstuffs by rumen microbes resulted in the concentration of NH_3_ required for microbial protein synthesis. Our results are lower in comparison with a previous study by Mitsumori *et al.*, [46] which was 9.99 mg/100 mL [46], while the concentration of NH_3_ in Bali cattle is a bit higher than that in a previous study by Pamungkas *et al*. [47] (14.24–27.91 mg/100 mL).

The concentration of acetic acid was significantly influenced (p < 0.05) by the breed of cattle, where the highest acetic acid concentration was in Bali cattle, followed by Madura and OC cattle. The concentration of acetic acid in Madura cattle in the previous study by Umar *et al*. [38] was 72.68 mMol, in OC cattle was 77.65 mM [38], whereas the concentration of acetic acid in Bali cattle ranged from 57.77 to 73.28 mMol [48]. In ruminants, acetic acid is used as an energy source, as well as a precursor for milk fat formation, and is non-glucogenic in animal tissues [49].

The propionic acid concentration was significantly influenced (p < 0.05) by breed. Our results supported a previous report showing different propionic acid concentrations between breeds [43]. The highest propionic acid concentration was observed in Bali cattle, followed by Madura and OC cattle. The effect of breed on propionic acid concentration was also reported by Bharanidharan *et al*. [42]. In a previous study by Shaffer *et al*. [32], the propionic acid concentration in Madura cattle was 35.80 mMol, whereas that in OC cattle was 44.14 mMol [38]. Propionic acid is the main precursor for the formation of blood glucose, and it is glucogenic [49]. The high propionic acid concentration in Bali cattle is expected to impact the highest productivity of Bali cattle. Propionic acid can produce and store more energy because it is glucogenic. The present study showed that the lowest ratio of acetic acid to propionic acid was in Bali cattle, so it is expected that Bali cattle might produce less methane gas emissions from fermentation in the digestive tract [46] and produce more meat than Madura and OC cattle. In this sense, Bali cattle use feed more efficiently. This has been shown by previous studies on feed efficiency that feed efficiency in Bali, OC, and Madura cattle were 9.29%–14.14% [50], 2% [51] to 13.55%–14% [52], and 5.54%–9.83% [53], respectively.

**Figure 2:**
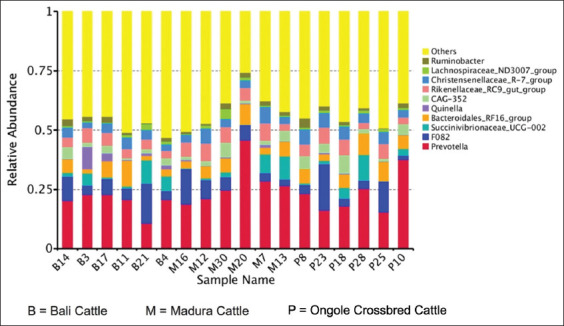
Dominant genera of rumen bacteria from each experimental animal.

The 10 dominant genera found in the rumen of three indigenous Indonesian cattle were *Ruminobacter*, *Lachnospiraceae*_ND3007_group, and *Christensenellaceae R-7-group*. *Rikenellacea_RC9_gut_group*, *CAG_352, Quinella, Bacteroidales_RF16_group, Succinivibrionaceae_UCG-002, F082*, and *Prevotella* (Figure 2). There was a type of dominant bacteria (*Quinella*) in the rumen fluid of Bali cattle, but it was almost not found in Madura and OC cattle. *Saccunaclasticum* is a propionate-producing bacterium, and *Bacteriodales* is a VFA producer [42]. Many *Quinella* populations contribute to low methane production [54]. The relatively large amount of *Saccunaclasticum* was probably caused by the high concentration of the concentrate offered. Several reports have also shown the effects of breed on rumen fermentation parameters [42, 43, 55]. Furthermore, there was a significant correlation between the type of rumen bacteria and serum biochemical parameters, rumen pH, and rumen fermentation patterns [42, 55, 56].

The concentration of total VFA was significantly influenced by the breed of cattle, where the highest concentration of total VFA was observed in Bali cattle, followed by Madura and OC cattle. The normal range of total VFA is 70–150 mMol/L [55], whereas another study reported that the production of total VFA in Bali cattle fed several levels of energy: protein balance ranged from 161.08–170.32 mMol/L [48]. In this study, the total VFA in Bali cattle was in a normal recommendation range but was lower than that reported by Vlaeminck *et al*. [49].

### Blood cell profile

The results of this study indicated that the number of RBCs was influenced by the breed of the cattle. The highest number of RBCs is observed in OC cattle [57]. Kim *et al*. [58] reported that beef cattle have higher RBC counts than dairy cattle [59], bulls have greater RBC counts than cows, and non-lactating cows have higher RBC counts than lactating cows [58]. The numbers of erythrocytes obtained in the OC, Bali, and Madura cattle were in the normal range, which indicated that the cattle had a total of 5.0–10.0 × 10^6^ erythrocytes per µL [60–62]. This indicates that the animal nutrient requirement was met.

In this study, blood hematocrit values were influenced by breed. There are differences in hematocrit values between Aceh and Bali cattle [60]. Furthermore, Etim [63] stated that age, sex, breed, and management systems are among the factors that influence the blood-based parameters of farm animals. The highest hematocrit was in Bali cattle and the lowest in OC cattle; however, the hematocrit values in these three breeds were still in the normal recommended range of hematocrit values for cattle, which is 21%–33% [59, 61]. A high hematocrit value indicates dehydration and venous polycythemia. An increase in the hematocrit value occurs due to a decrease in blood plasma volume under dehydration conditions; thus, the ratio of RBC to blood plasma is above normal. The anemia symptoms based on the percentage of hematocrit were classified as mild anemia (20%–26%), moderate anemia (14%–19%), severe anemia (10%–13%), and very severe anemia (10%–13%) [64, 65]. Dehydration in cattle is also attributed to insufficient nutrition and an inadequate environment [61, 66]. Dehydration in cattle adversely affects, especially milk yield and reproductive efficiency and causes economic losses by causing deaths [59, 65, 67–69].

The normal value of MCH in cattle is 11–17 pg, whereas the range of MCV in cattle is 40.0–60.0 fl. [70]. All cattle breeds were in the normal recommended range for MCH and MCV, except for OC cattle, which had a slightly lower rate thanpreviously reported by Widayati *et al*. [40].

The MCH value in this study was still in the normal range for cattle, although the breed of the cattle showed significant differences. The Bali cattle had the highest MCH, followed by the Madura and OC cattle. A low MCV value indicates microcytic anemia caused by Fe and Cu deficiency, worm infection, or impaired Fe absorption [60]. The MCV values in Bali and Madura were normal. High and low MCV values in cattle are also influenced by several factors, such as the nutrients in the feed (Fe, Cu, amino acids, Vitamin B9, and Vitamin B12) that affect the number and shape of erythrocytes.

## CONCLUSION

This study provides valuable insights into the ruminal ecology and blood profiles of three predominant indigenous Indonesian cattle breeds namely Bali, Madura, and OC. The findings revealed that Bali cattle exhibited superior rumen fermentation profiles, including the highest concentrations of total volatile fatty acids (139.66 mMol) and propionic acid (33.31 mMol), along with a lower acetic-to-propionic acid ratio. These traits indicate their efficient glucogenic metabolism and potential for higher productive performance. In terms of blood profiles, OC cattle demonstrated the highest RBC counts (9.27 × 10³/µL), while Bali cattle displayed better hematological indices, including the largest RBC size (MCV: 48.84 fl) and highest hemoglobin content (MCH: 16.60 pg). The unique presence of *Quinella*, a propionate-producing bacterium, in Bali cattle further highlights their metabolic efficiency.

The study provides a comprehensive comparison of three cattle breeds, leveraging robust experimental design and statistical methods. It integrates biochemical, microbiological, and physiological analyses to offer a holistic view of breed-specific traits. However, it focused on a relatively small sample size and was conducted under controlled conditions, which may not fully represent the variability seen in traditional farming systems. In addition, seasonal and dietary variations, which could influence ruminal and hematological profiles, were not extensively explored.

Further research should focus on evaluating these breeds under diverse feeding systems and environmental conditions to validate the findings. Exploring the genetic basis of ruminal fermentation efficiency and blood profiles could provide deeper insights. In addition, studies aimed at correlating these traits with productive performance, methane emissions, and overall sustainability in smallholder systems will help develop targeted breeding and management strategies. This study highlights the potential of Bali cattle for sustainable meat production, given their superior metabolic traits, and lays the groundwork for optimizing breed-specific strategies to enhance livestock productivity in tropical regions.

## AUTHORS’ CONTRIBUTIONS

All authors contributed to the formulation and conceptualization of the study design. YNA, PWP, MM, RA, DP, BT, YW, MNR, WN, and ESR: Conceptualization. YNA, PWP, MNA, MM, BT, YW, MNR, and WN: Methodology. YNA, MNA, RA, NHK, MNR, WN, SS, ESR, WTS, and FF: Software. YNA, PWP, MNA, MM, RA, NHK, DP, BT, MNR, and WN: Investigation, Validation, and formal analysis.YNA, PWP, MNA, MM, RA, NHK, DP, YW, WN, MNR, SS, ESR, FF, and WTS: Data curation and visualization. YNA, PWP, MNA, RA, NHK, DP, BT, WN, ESR, FF, and WTS: Writing-original draft preparation. YNA, MM, RA, NHK, DP, BT, SS, FF, and WTS: Writing-review and editing. All authors have read and approved the final manuscript.
